# Identification of SARS1 as a Prognostic Biomarker in Invasive Lobular Carcinoma Using Lasso-Logistic Regression

**DOI:** 10.17912/micropub.biology.001688

**Published:** 2025-08-18

**Authors:** Tyrel Porter, Isaí Soto-Colón, Emir Rinaldi-Pérez, David Rivera-Aponte

**Affiliations:** 1 School of Medicine, Universidad Central del Caribe, Bayamón, Puerto Rico; 2 Department of Biochemistry, Universidad Central del Caribe, Bayamón, Puerto Rico

## Abstract

Aminoacyl-tRNA synthetases (ARSs) have emerging roles in cancer biology. Specifically, *TARS1* has been linked to immunosuppressive microenvironments and poor prognosis in breast cancer. To identify other ARS genes with prognostic relevance in invasive lobular carcinoma (ILC), we applied lasso-logistic regression to 41 ARS genes using TCGA RNA-seq data and using patient vital status as the binary outcome. Survival analysis showed *SARS1* expression was associated with significantly reduced survival across 5 and 10 years, while *CARS2* showed significance at 5 years only. These findings support *SARS1* as a candidate prognostic biomarker in ILC and suggest broader relevance for ARS genes in breast cancer outcomes.

**Figure 1. Identification and prognostic evaluation of aminoacyl-tRNA synthetase genes in invasive lobular carcinoma:**
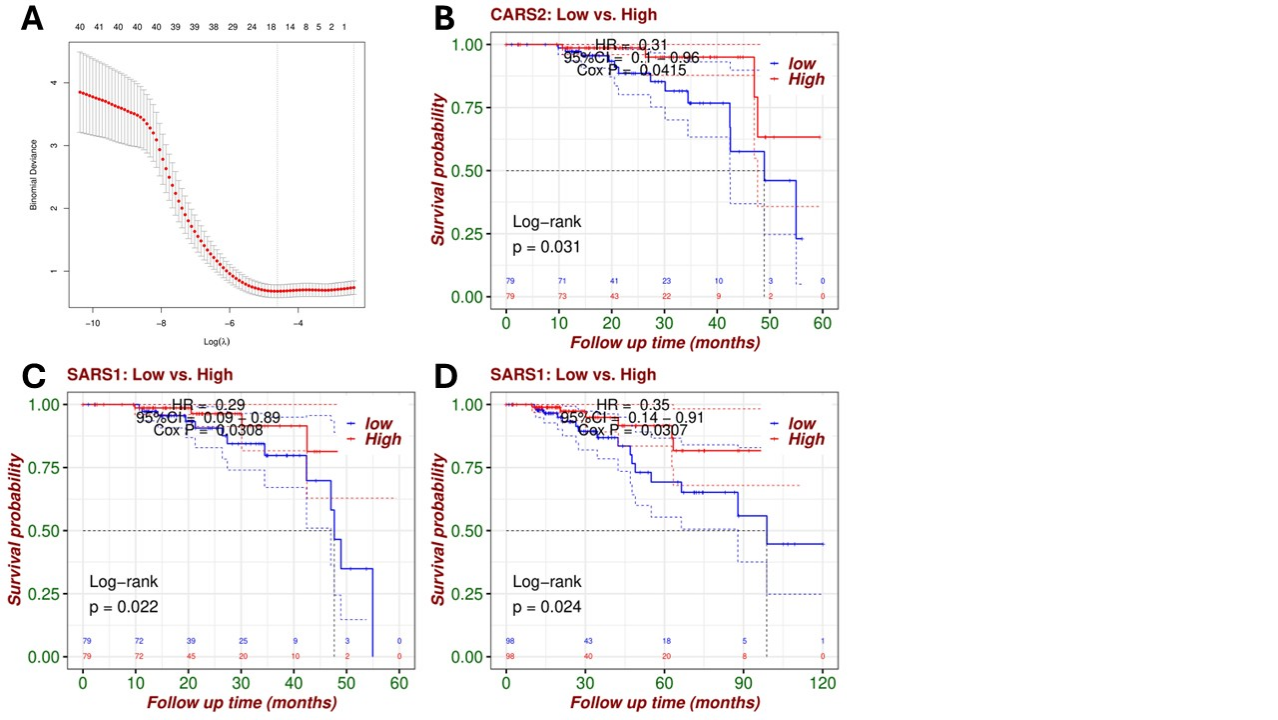
(A) Lasso-logistic regression plot showing coefficient shrinkage. Model was chosen to identify genes associated with patient survival status by comparing deceased vs. living groups, ultimately yielding nine genes with nonzero coefficients.(B) Kaplan–Meier curve for 5-year overall survival stratified by median expression of ***CARS2* .** High expression was associated with improved survival (HR = 0.31, 95% CI: 0.1–0.96, Cox *p* = 0.0415, log-rank *p* = 0.031).(C) Kaplan–Meier curve for 5-year overall survival stratified by ***SARS1*** expression. High expression correlated with significantly better survival (HR = 0.29, 95% CI: 0.09–0.89, Cox *p* = 0.0308, log-rank *p* = 0.022).(D) Kaplan–Meier curve for 10-year overall survival based on ***SARS1*** expression, reinforcing its long-term prognostic association (HR = 0.35, 95% CI: 0.14–0.91, Cox *p* = 0.0307, log-rank *p* = 0.024).

## Description

Aminoacyl-tRNA synthetases (ARS) are best known for their role in catalyzing the attachment of amino acids to tRNA molecules during protein synthesis. However, recent studies have uncovered their broader relevance in cancer biology, including roles in angiogenesis, immune regulation, and cell proliferation (Gupta et al., 2023; Sung et al., 2022; Park et al., 2008). *TARS1* , for example, has been associated with immunosuppressive tumor microenvironments in breast cancer, favoring infiltration of regulatory T cells and Th2 cells while limiting cytotoxic immune cell presence **(Gui et al., 2024)** . Additionally, this study showed that in breast cancer high *TARS1* expression has been linked to aggressive tumor features such as hormone receptor negativity and increased tumor size.

Invasive lobular carcinoma (ILC), the second most common histological subtype of breast cancer, presents unique clinical challenges due to its diffuse growth pattern and often subtle radiographic appearance. Despite its prevalence, molecular biomarkers specific to ILC remain limited and its distinct biology is often overshadowed in research by the more common invasive ductal carcinoma. Motivated by the precedent set by *TARS1* , this study sought to identify additional ARS genes associated with prognosis in ILC, with the goal of uncovering subtype-specific insights that might be obscured in broader breast cancer analyses.

The lasso-logistic model, tuned using deviance minimization, yielded an intercept of 11.0888, selecting nine ARS genes with nonzero coefficients: *CARS2* (1.0703), *HARS2* (0.0702), *IARS1* (1.2348), *IARS2* (0.256), *PARS2* (0.3132), *RARS2* (0.2746), *SARS1* (0.4659), *SARS2* (0.0622), and *WARS1* (0.4328) **( Figure 1A )** . These results supported possible contribution to survival in the ILC cohort for these candidate genes.

Among the gene signature, two genes emerged as potentially prognostic. Of the two, *CARS2* showed limited prognostic potential. At 5 years, it was associated with improved survival (HR = 0.31, 95% CI: 0.1–0.96, log-rank *p* = 0.031, Cox *p* = 0.0415) **( Figure 1B ).** However, significance was lost when evaluating survival at 10 years (HR = 0.4, 95% CI: 0.16–1.03, log-rank *p* = 0.051, Cox *p* = 0.0587). *SARS1* demonstrated the most consistent signal, with a 5-year hazard ratio (HR) of 0.29 (95% CI: 0.09–0.89, log-rank *p* = 0.022, Cox *p* = 0.0308) **( Figure 1C )** . Additionally, at 10 years, SARS1 remained significant in both analyses (HR = 0.35, 95% CI: 0.14–0.91, log-rank *p* = 0.024, Cox *p* = 0.0307), supporting durable prognostic value **( Figure 1D )** .

The remaining genes— *HARS2* , *IARS1* , *IARS2* , *PARS2* , *RARS2* , *SARS2* , and *WARS1* —did not reach statistical significance, though their selection by lasso-logistic regression suggests they could be relevant in multivariate or pathway-based models.

These findings point to *SARS1* as a candidate prognostic biomarker in ILC. Its consistent association with favorable long-term survival invites further investigation into its biological function and potential role in therapeutic stratification. Moreover, recent research has demonstrated that seryl-tRNA synthetase, encoded by the *SARS1* gene, acts as a tumor and metastasis suppressor in breast cancer by inhibiting Wnt signaling pathways and reducing β-catenin levels **(Jiang et. al., 2025)** , supporting our observations. *CARS2,* which did not exhibit as significant results comparatively, may also warrant additional investigation. This is particularly the case given its mitochondrial function, which may influence cancer metabolism or apoptosis.

The limitations of this study include its reliance on a single public dataset. Furthermore, mRNA expression was assessed in bulk tumor tissue, without adjustment for tumor purity or cell-type composition, which could confound gene-level associations. Nonetheless, the identification of *SARS1* and *CARS2* as survival-associated genes enriches our understanding of ARS family involvement in ILC and lays the groundwork for future validation in independent cohorts.

## Methods

Data Acquisition

RNA-sequencing data and clinical metadata for female patients with invasive lobular carcinoma (ILC) were obtained from The Cancer Genome Atlas (TCGA). mRNA expression values were retrieved through the expOmics platform (Li et al., 2024), while clinical survival data were collected via cBioPortal (Cerami et al. 2012; Gao et al., 2013; de Bruijn et al., 2023). Only primary tumor samples were included, resulting in a final sample size of 211 patients. Expression values were normalized as transcripts per million (TPM).

Construction of Gene Signature

We focused on 41 genes annotated in the KEGG aminoacyl-tRNA biosynthesis pathway (KEGG pathway hsa00970). These include cytoplasmic and mitochondrial ARS genes with known or suspected relevance in cancer. A least absolute shrinkage and selection operator (lasso) logistic regression was performed using the FeatureSelectToolkit on expOmics, with relevant R packages listed in the original publication by (Li et al., 2024), with model tuning based on deviance to optimize feature selection. For this analysis, patient outcomes were dichotomized into living versus deceased at the study endpoint, allowing lasso logistic regression to identify genes potentially associated with survival. Lasso prioritizes candidate genes for further analysis but does not confirm statistical significance on its own. Therefore, candidate genes were then subjected to individual survival analyses to confirm their association with patient outcomes.

Survival Analysis

Kaplan-Meier survival curves were generated for each gene with a nonzero lasso-logistic coefficient. Patients were dichotomized into high and low expression groups based on the median expression value of each gene. Differences in survival distributions between groups were assessed using the log-rank test, while Cox proportional hazards models were used to estimate hazard ratios (HRs) and corresponding *p* -values. Both analyses were performed using GraphPad Prism v10.5.0 and expOmics. Survival was evaluated at 5-year and 10-year time points, and statistical significance was defined as *p* < 0.05.

## References

[R1] Cerami E, Gao J, Dogrusoz U, Gross BE, Sumer SO, Aksoy BA, Jacobsen A, Byrne CJ, Heuer ML, Larsson E, Antipin Y, Reva B, Goldberg AP, Sander C, Schultz N (2012). The cBio cancer genomics portal: an open platform for exploring multidimensional cancer genomics data.. Cancer Discov.

[R2] de Bruijn I, Kundra R, Mastrogiacomo B, Tran TN, Sikina L, Mazor T, Li X, Ochoa A, Zhao G, Lai B, Abeshouse A, Baiceanu D, Ciftci E, Dogrusoz U, Dufilie A, Erkoc Z, Garcia Lara E, Fu Z, Gross B, Haynes C, Heath A, Higgins D, Jagannathan P, Kalletla K, Kumari P, Lindsay J, Lisman A, Leenknegt B, Lukasse P, Madela D, Madupuri R, van Nierop P, Plantalech O, Quach J, Resnick AC, Rodenburg SYA, Satravada BA, Schaeffer F, Sheridan R, Singh J, Sirohi R, Sumer SO, van Hagen S, Wang A, Wilson M, Zhang H, Zhu K, Rusk N, Brown S, Lavery JA, Panageas KS, Rudolph JE, LeNoue-Newton ML, Warner JL, Guo X, Hunter-Zinck H, Yu TV, Pilai S, Nichols C, Gardos SM, Philip J, Kehl KL, Riely GJ, Schrag D, Lee J, Fiandalo MV, Sweeney SM, Pugh TJ, Sander C, Cerami E, Gao J, Schultz N, AACR Project GENIE BPC Core Team, AACR Project GENIE Consortium (2023). Analysis and Visualization of Longitudinal Genomic and Clinical Data from the AACR Project GENIE Biopharma Collaborative in cBioPortal.. Cancer Res.

[R3] Gao J, Aksoy BA, Dogrusoz U, Dresdner G, Gross B, Sumer SO, Sun Y, Jacobsen A, Sinha R, Larsson E, Cerami E, Sander C, Schultz N (2013). Integrative analysis of complex cancer genomics and clinical profiles using the cBioPortal.. Sci Signal.

[R4] Gupta S, Jani J, Vijayasurya, Mochi J, Tabasum S, Sabarwal A, Pappachan A (2023). Aminoacyl-tRNA synthetase - a molecular multitasker.. FASEB J.

[R5] Gui Z, Liu P, Zhang D, Wang W (2023). Clinical implications and immune implications features of TARS1 in breast cancer.. Front Oncol.

[R6] Jiang L, Wang J, Liu Z, Zhang Q, Yang XL (2025). Seryl-tRNA synthetase inhibits Wnt signaling and breast cancer progression and metastasis.. FASEB J.

[R7] Li D, Min Z, Guo J, Chen Y, Zhang W (2024). ExpOmics: a comprehensive web platform empowering biologists with robust multi-omics data analysis capabilities.. Bioinformatics.

[R8] Park SG, Schimmel P, Kim S (2008). Aminoacyl tRNA synthetases and their connections to disease.. Proc Natl Acad Sci U S A.

[R9] Sung Y, Yoon I, Han JM, Kim S (2022). Functional and pathologic association of aminoacyl-tRNA synthetases with cancer.. Exp Mol Med.

